# Protective versus pathologic pre-exposure cytokine profiles in dengue virus infection

**DOI:** 10.1371/journal.pntd.0006975

**Published:** 2018-12-17

**Authors:** Heather Friberg, Coreen M. Beaumier, Sangshin Park, Pamela Pazoles, Timothy P. Endy, Anuja Mathew, Jeffrey R. Currier, Richard G. Jarman, Kathryn B. Anderson, Steven Hatch, Stephen J. Thomas, Alan L. Rothman

**Affiliations:** 1 Viral Diseases Branch, Walter Reed Army Institute of Research, Silver Spring, Maryland, United States of America; 2 Center for International Health Research, Rhode Island Hospital, The Warren Alpert Medical School of Brown University, Providence, Rhode Island, United States of America; 3 Department of Pediatrics, The Warren Alpert Medical School of Brown University, Providence, Rhode Island, United States of America; 4 Division of Infectious Diseases and Immunology, Department of Medicine, University of Massachusetts Medical School, Worcester, Massachusetts, United States of America; 5 Department of Virology, Armed Forces Research Institute of Medical Sciences, Bangkok, Thailand; 6 Institute for Immunology and Informatics, Department of Cell and Molecular Biology, University of Rhode Island, Providence, Rhode Island, United States of America; 7 Department of Medicine, University of Minnesota, Minneapolis, Minnesota, United States of America; University of Texas Medical Branch, UNITED STATES

## Abstract

**Background:**

Hyperendemic circulation of all four types of dengue virus (DENV-1-4) has expanded globally, fueling concern for increased incidence of severe dengue. While the majority of DENV infections are subclinical, epidemiologic studies suggest that type-cross-reactive immunity can influence disease outcome in subsequent infections. The mechanisms controlling these differential clinical outcomes remain poorly defined.

**Methodology/Principal findings:**

Blood samples were collected from a cohort of school-aged Thai children who subsequently experienced a subclinical DENV infection or developed dengue illness. PBMC collected prior to infection were stimulated *in vitro* with DENV and the secretion of 30 cytokines was measured using a multiplexed, bead-based array. Significant differences were found in cytokine production based on both the type of DENV used for stimulation and the occurrence of clinical illness. Secretion of IL-15 and MCP-1 was significantly higher by PBMC of subjects who later developed symptomatic DENV infection. In addition, IL-6 was produced by PBMC from all subjects who subsequently developed symptomatic infection, versus 59% of subjects who had subclinical infection. Secretion of IL-12, IL-2R, MIP-1α, RANTES, GM-CSF, and TNFα was significantly lower by PBMC from subjects with symptomatic infection.

**Conclusions/Significance:**

These data demonstrate significant differences in pre-existing immune responses to DENV associated with the clinical outcome of subsequent infection. The finding of higher levels of some cytokines in subjects with symptomatic infection and higher levels of other cytokines in subjects with subclinical infection supports the existence of both protective and pathologic immune profiles. Clinical-immunological correlations identified in the context of natural DENV infection may be useful for evaluating immune responses to dengue vaccines.

## Author summary

Dengue is one of the most prevalent mosquito-borne infectious diseases worldwide. It is caused by one of four viruses, types 1–4, and ranges in severity from subclinical (mild or no symptoms) to dengue fever (febrile illness with headache and bone pain), or less frequently, dengue hemorrhagic fever, where patients experience leaky blood vessels, sometimes significant bleeding, and may be severe enough to cause death. While many risk factors have been associated with development of severe dengue, sequential infection with different virus types is a major factor, implying that the immune response generated after an initial infection is partly responsible for making subsequent exposure clinically worse. This study sought to identify profiles of immune markers that correlate with increased or decreased risk of dengue. Using samples from individuals ~5 months prior to dengue virus infection, who later experienced either subclinical infection or overt disease, we modeled virus exposure *in vitro* and compared the production of various immune proteins between the two outcome groups. Three of the proteins studied were produced at higher levels by subjects who subsequently had dengue, and six of the proteins were produced at higher levels by subjects who subsequently had subclinical infection. These results help define what constitutes beneficial versus potentially harmful immune responses, aiding in the design of effective dengue vaccines.

## Introduction

Infection with any of the four types of dengue virus (DENV-1-4) can lead to a range of outcomes, from asymptomatic or subclinical infection to classical dengue fever (DF), dengue hemorrhagic fever (DHF), or, the most severe outcome, dengue shock syndrome (DSS). The majority of DENV infections have been shown to result in subclinical outcomes [1, 2]. Patients with DHF/DSS are most often infants born to DENV-immune mothers or individuals experiencing a secondary DENV infection, indicating a role for pre-existing DENV immunity in exacerbating dengue disease [3]. A major challenge for researchers has been identifying the specific immunologic factor(s) that contribute individually or in concert to dengue pathology or protection from disease.

Amongst the immune markers studied, higher levels of particular cytokines and chemokines have been found in the sera of subjects with severe dengue compared to mild disease. A number of pro-inflammatory cytokines in particular have been implicated in severe dengue, including TNFα, IFNγ, IL-18, IL-1β, and IL-6 [4–6]. In most of those studies, sera or PBMC obtained during or after secondary DENV infection were tested. PBMC obtained prior to infection have typically not been available to determine whether pre-existing immune profiles could predict whether an individual developed subsequent subclinical infection or severe dengue.

Through a unique cohort study in Thailand, school-age children were subjected to active, school absence-based surveillance, and routine blood samples were obtained before and after the peak DENV transmission season [1, 7, 8]. The study design allowed detection of DENV seroconversions, i.e., a four-fold or greater increase in anti-DENV antibody titer between pre- and post-season blood draws, in the presence or absence of overt, clinically relevant illness (referred to hereafter as symptomatic and subclinical infections, respectively). In previously published studies of this school-based cohort, memory T cell responses in pre-exposure PBMC were evaluated. Higher frequencies of DENV–specific IFNγ- and IL-2-producing T cells were reported among schoolchildren who subsequently developed subclinical infection, compared with those who developed symptomatic secondary DENV infection [9]. Those data suggested a link between cytokine-producing T cells and subsequent mild or subclinical illness. In the current study, we extended our analysis of cytokine production associated with the clinical outcome of subsequent DENV infection by stimulating pre-infection PBMC samples with live virus and evaluating cytokine profiles in cell supernatants using a multiplexed cytokine immunoassay.

## Methods

### Ethics statement

Written informed consent was obtained from the parents/guardians of all subjects, and the clinical investigation was conducted according to the principles expressed in the Declaration of Helsinki. These studies were approved by the institutional review boards at the University of Massachusetts Medical School, the Ministry of Public Health in Thailand, and the Human Subjects Research Review Board for the Commanding General of the US Army Medical Research and Material Command.

### Specimens

Blood specimens were obtained from a prospective cohort of school-aged children in Kamphaeng Phet, Thailand, which has been described previously [1, 7, 8]. Briefly, plasma samples were collected from enrolled subjects in January, June, August, and November and tested by hemagglutination inhibition (HI) assay. A ≥4-fold increase in anti-DENV HI titer between the January sample (pre) and any of the subsequent routine samples (post) was used to identify seroconversions. Study participants were also monitored for school absences associated with fever from June to November. Seroconversions which occurred without school-related absenteeism were defined as subclinical infections, and those which occurred with school-related absenteeism and which were associated with symptomatic laboratory-confirmed DENV infections during the corresponding period were defined as symptomatic infections. Clinical illnesses were classified according to the 1997 WHO guidelines [10]. DENV infection was defined by virus isolation and/or detection of virus by reverse transcriptase polymerase chain reaction (RT-PCR) in acute serum samples and dengue IgM/IgG enzyme immunoassay (EIA) or HI assays against all 4 DENV types, as previously described [7]. Primary infection was defined by an IgM-to-IgG ratio of ≥1.8 by EIA in the early convalescent sera obtained from symptomatic individuals [7]. Plaque reduction neutralization test (PRNT) assays were performed as previously described [11] on all subjects with positive HI titers.

### *In vitro* stimulation and culture

Cryopreserved PBMC were shipped from Thailand on dry ice to the US and stored in liquid nitrogen until testing. Vials of PBMC were thawed and counted. PBMC (100,000 cells/well) were seeded into a 96-well plate in 200 μL/well RPMI 1640 medium (Gibco, Carlsbad, CA, USA) supplemented with 10% fetal bovine serum (FBS; Sigma Immunochemicals, St. Louis, MO, USA), 200 mM L-glutamine (Gibco), and 100 U/mL penicillin/streptomycin (Gibco) and stimulated with 1:1000 anti-CD3 (clone 12F6; a gift generously provided by Dr. Johnson Wang), 1.2 μL/well uninfected Vero cell supernatant (negative control), or live DENV-1, live DENV-2, live DENV-3, or live DENV-4, each at a multiplicity of infection (MOI) of 1. The viruses used for stimulation were DENV-1 strain WP-74 (titer, 6.96 x 10^6^ plaque-forming units [PFU]/mL), DENV-2 strain S16803 (titer, 1.4 x 10^9^ PFU/mL), DENV-3 strain CH53489 (titer, 8.6 x 10^7^ PFU/mL), and DENV-4 strain 341750 (titer, 5.8 x 10^8^ PFU/mL), all of which were propagated in Vero cells. Each stimulation condition was performed in triplicate. The stimulated PBMC were maintained in culture for 6–7 days, at which time the supernatants were harvested, spun down to clear debris, and frozen at -20°C until analysis.

### Cytokine analysis

The Human Cytokine 30-plex Panel kit (LHC6003, Invitrogen, Life Technologies, Grand Island, NY, USA) was used to determine the concentration of 30 different cytokines and chemokines present in the tissue culture supernatant samples, including EGF, Eotaxin, FGF-basic, G-CSF, GM-CSF, HGF, IFNα, IFNγ, IL-1RA, IL-1β, IL-2, IL-2R, IL-4, IL-5, IL-6, IL-7, IL-8, IL-10, IL-12 (p40/p70), IL-13, IL-15, IL-17, IP-10, MCP-1, MIG, MIP-1α, MIP-1β, RANTES, TNFα, and VEGF. The kit was used according to the manufacturer’s instructions. Briefly, the frozen tissue culture samples were thawed, plated undiluted (in duplicate) onto 96-well MultiScreen HTS filter plates (MSBVS1210; EMD Millipore Corp., Billerica, MA, USA) containing 30-plex, antibody-coated beads, and incubated on a shaker platform (MixMate; Eppendorf, Hauppauge, NY, USA) at 550 rpm for 2 hours. The wells were then washed using a MultiScreen HTS Vacuum Manifold (Millipore), and the analyte-bound beads were tagged with biotin-conjugated antibodies, washed, and visualized with streptavidin-conjugated phycoerythrin (PE). The data were acquired on a Luminex 200 instrument (Luminex Corp., Austin, TX, USA) and analyzed using Luminex 100 Integrated System 2.3 software (Luminex Corp.). Protein standards were provided in the kit, and standard curves were generated with eight standard dilutions (undiluted, 1:3, 1:9, 1:27, 1:81, 1:243, 1:729, 1:2187) in RPMI/10% FBS.

### Intracellular cytokine staining (ICS)

ICS assays were performed in parallel with the *in vitro* stimulation and culture assays described above. Cryopreserved PBMC were thawed, counted, and approximately 0.2-1x10^6^ viable cells (median, 0.5x10^6^; the number of cells varied from subject-to-subject but were the same for all stimulation conditions for a given subject) were plated into each well of a 96-well plate. Cells were stimulated with a 1:40 dilution of inactivated antigen (supernatant from glutaraldehyde-treated and lysed Vero cells uninfected [negative control] or infected with DENV-1, DENV-2, DENV-3, or DENV-4, as previously described [12]) in complete RPMI/10% FBS medium at 37°C. After overnight incubation (approximately 12 hours), protein transport inhibitors (Golgi Plug/Stop, BD Biosciences) were added and the cells incubated at 37°C for an additional 6 hours. The cells were then washed and stained with LIVE/DEAD Aqua (Invitrogen) before fixing and permeabilizing in CytoFix/CytoPerm (BD Biosciences), resuspending in Perm Wash Buffer (BD Biosciences), and adding the cells to a BD Lyoplate (623668; BD Biosciences). The Lyoplate contained a pre-mixed cocktail of antibodies: anti-CD3-V450, anti-IFNγ-Alexa Fluor 488, anti-IL-2-PE, anti-CD4-PerCP-Cy5.5, anti-TNFα-PE-Cy7, and anti-CD8-APC-H7. The cells were incubated with the antibody cocktail at room temperature for 30 minutes. Data were acquired on an LSR II flow cytometer (BD Biosciences) and analyzed using FlowJo v9 software (Tree Star, Inc.).

### Statistical analysis

Standard curves were generated for each of the 30 analytes using a five-parameter logistic curve fit and 1/y^2^ weighted function by the Luminex 100 Integrated System 2.3 software. The data were then exported and statistically analyzed using SAS 9.4 software (SAS Institute, Cary, NC). The non-parametric Wilcoxon rank-sum test was used to compare cytokine production between subjects with subclinical and symptomatic infections. A *P* value <0.05 was considered to be statistically significant.

## Results

We identified 51 subjects who had DENV seroconversions during 1998 for this study, as determined by a 4-fold rise in HI titer to at least one DENV type (Table 1). Of these, 29 subjects experienced no overt clinical illness (subclinical infections) and 22 had illnesses that resulted in school absenteeism (symptomatic infections), 3 of which were hospitalized. No significant differences were found between subjects who experienced subclinical versus symptomatic infections with regard to age (mean [range], 10.2 [8–13] years and 9.5 [7–12] years, respectively) or sex (M:F ratio, 1.07 and 1.75, respectively). While no blood samples were available from subjects with subclinical infections at the time of exposure, acute samples from those with symptomatic infections showed a predominance of infection with DENV-3 followed by DENV-1; a single DENV-2 infection was detected and no DENV-4 infections. Five samples were negative by PCR, therefore the infecting DENV type is not known for those subjects.

**Table 1:** Characteristics of the study population.

Subject No.	HI Titer (pre)^a^				HI Titer (post)^b^				Clinical Outcome	DV Type (PCR)
	DV-1	DV-2	DV-3	DV-4	DV-1	DV-2	DV-3	DV-4		
1	<10	<10	<10	<10	40	40	80	80	Subclinical	--
2	<10	<10	<10	<10	20	40	40	40	Subclinical	--
3	<10	<10	10	<10	1280	1280	1280	2560	Subclinical	--
4	<10	<10	<10	<10	320	640	320	20481	Subclinical	--
5	10	<10	10	10	40	20	40	40	Subclinical	--
6	10	20	10	10	20	40	40	40	Subclinical	--
7	<10	<10	<10	<10	10	20	80	20	Subclinical	--
8	<10	<10	<10	<10	320	320	640	640	Subclinical	--
9	<10	<10	<10	<10	1280	640	1280	640	Subclinical	--
10	<10	<10	<10	<10	80	80	80	80	Subclinical	--
11	<10	<10	10	<10	320	640	1280	1280	Subclinical	--
12	<10	<10	<10	<10	80	80	80	320	Subclinical	--
13	10	10	20	10	1280	640	640	640	Subclinical	--
14	<10	<10	<10	<10	640	1280	2560	1280	Subclinical	--
15	20	20	<10	<10	160	320	320	80	Subclinical	--
16	<10	<10	10	10	40	40	40	40	Subclinical	--
17	<10	<10	10	<10	320	320	320	320	Subclinical	--
18	<10	<10	<10	<10	640	320	640	640	Subclinical	--
19	<10	10	<10	<10	160	320	320	320	Subclinical	--
20	<10	<10	<10	<10	1280	2560	2560	5120	Subclinical	--
21	10	40	10	10	160	320	320	320	Subclinical	--
22	<10	20	10	10	40	40	80	80	Subclinical	--
23	<10	<10	<10	<10	20	<10	40	20	Subclinical	--
24	<10	<10	<10	10	160	160	640	640	Subclinical	--
25	<10	<10	<10	<10	40	20	40	80	Subclinical	--
26	<10	<10	10	10	10	<10	20	40	Subclinical	--
27	<10	10	20	20	<10	<10	80	160	Subclinical	--
28	10	<10	10	10	10	10	20	40	Subclinical	--
29	<10	<10	<10	<10	20	20	40	40	Subclinical	--
30	<10	<10	<10	<10	80	80	160	160	DF	DV-1
31	<10	<10	<10	<10	640	320	640	320	DF	DV-1
32	<10	<10	<10	<10	640	1280	1280	1280	DF	Neg
33	<10	<10	10	10	1280	1280	640	1280	DF	DV-3
34	<10	<10	<10	<10	320	320	640	1280	DF	DV-3
35	10	<10	10	<10	1280	640	640	320	DF	Neg
36	20	10	<10	10	2560	1280	2560	1280	DF	Neg
37	<10	<10	<10	<10	160	320	640	320	DF	DV-1
38	<10	<10	<10	<10	160	160	1280	640	DF	Neg
39	<10	<10	10	10	320	320	1280	1280	DF	DV-3
40	<10	<10	20	<10	80	80	640	160	DF	DV-3
41	<10	<10	<10	<10	320	640	640	640	DF	DV-3
42	<10	<10	<10	<10	640	1280	1280	1280	DF	DV-2
43	<10	<10	<10	<10	80	20	40	40	DF	DV-1
44	<10	<10	<10	<10	640	320	640	640	DF	DV-3
45	<10	20	<10	<10	1280	1280	2560	1280	DF	DV-3
46	<10	<10	<10	<10	1280	1280	1280	1280	DF	DV-3
47	<10	10	<10	<10	1280	1280	2560	1280	DF	DV-3
48	<10	<10	<10	<10	160	160	320	320	DF	Neg
49	<10	<10	<10	<10	640	640	1280	2560	DHFIII	DV-3
50	20	20	10	10	640	1280	2560	640	hDF	DV-3
51	<10	<10	<10	10	640	320	1280	1280	hDF	DV-1

^a^Prior to the 1998 season (January sample)

^b^First sample from a scheduled visit (June, August, or November) that showed a 4-fold rise in HI titer relative to the January sample

Abbreviations: DF, dengue fever; DHFIII, dengue hemorrhagic fever grade 3; DV-1-4, dengue virus types 1–4; hDF, hospitalized DF; HI, hemagglutination inhibition; Neg, negative

DENV-specific neutralizing antibodies (NAb) were undetectable in 22 subjects prior to the 1998 dengue season (S1 Table). However, NAb profiles assessed after the dengue season were predominantly multi-typic, characteristic of a secondary-type antibody response. In symptomatic subjects, IgM/IgG EIA titers also indicated the occurrence of secondary infections in all but one case, despite some of the subjects having no anti-DENV NAb titers at the beginning of the year. These results indicate that most subjects had prior exposure to DENV.

PBMC were collected from all subjects in January, prior to the dengue season, and cryopreserved. The time interval between the pre-exposure sample and DENV infection varied from subject-to-subject, but on average was 163 (range, 121–207) days to the first day of illness for symptomatic subjects. The time interval between the pre-exposure sample and the first sample with a positive HI titer was on average 218 (range, 106–306) days for subclinical subjects and 189 (range, 119–280) days for symptomatic subjects. This suggests subclinical infections occurred on a similar timescale as symptomatic infections.

**Fig 1:**
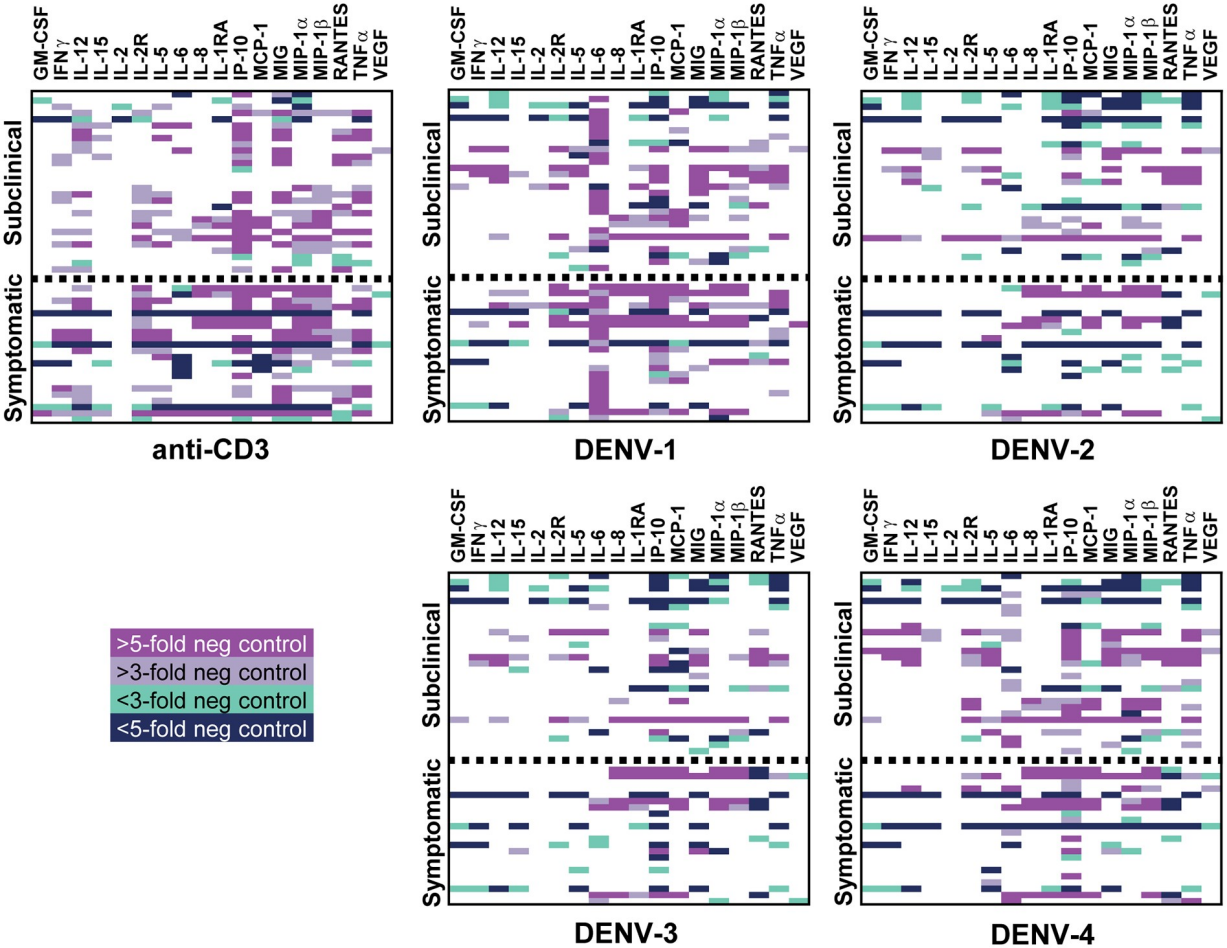
Relative expression of cytokines in cell culture supernatants from stimulated pre-illness PBMC. PBMC from subjects who went on to experience subclinical (n = 29) or symptomatic (n = 22) DENV infections were stimulated *in vitro* with anti-CD3 antibody (positive control), live DENV-1, live DENV-2, live DENV-3, live DENV-4, or uninfected Vero cell supernatant (negative control). After 6–7 days, culture supernatants were assessed by a multiplexed, bead-based array for quantification of 30 cytokines/chemokines/growth factors. Shown are three- and five-fold changes up (light and dark blue, respectively) or down (grey and pink, respectively), relative to the negative control, of each listed analyte. Each row represents responses from a single individual.

For this study, we wanted to compare the cell-derived cytokine profiles elicited from those who went on to experience subclinical versus symptomatic DENV infections to determine whether particular profiles could predict subsequent clinical outcome. To do this, the pre-exposure PBMC samples were thawed and placed in separate cultures with each of the four types of (live) DENV. The tissue culture supernatants were collected after 1 week and subjected to a multiplexed analysis of 30 different cytokines, chemokines, and growth factors (Fig 1 and S1 Fig).

Using this assay format we found undetectable or very low levels of EGF, Eotaxin, FGF-basic, G-CSF, IL-1β, and IL-4 overall, and minimal DENV-specific production (detected in five or fewer subjects, <10% of the study cohort) of HGF, IFNα, IL-7, IL-10, IL-13, and IL-17 (S1 Fig). IL-2 was also only detectable in a handful of subjects (Fig 1), but this may reflect its consumption during the culture by cells responding to DENV stimulation. While PBMC from many subjects responded to DENV stimulation with production of IL-5, IL-8, IL-1RA, IP-10, and MIG, no correlations were observed with respect to clinical outcome (Fig 1). The remaining 12 cytokines showed patterns that appeared to differ by clinical outcome.

### Increased production of cytokines in subjects with subclinical versus symptomatic infections

**Fig 2:**
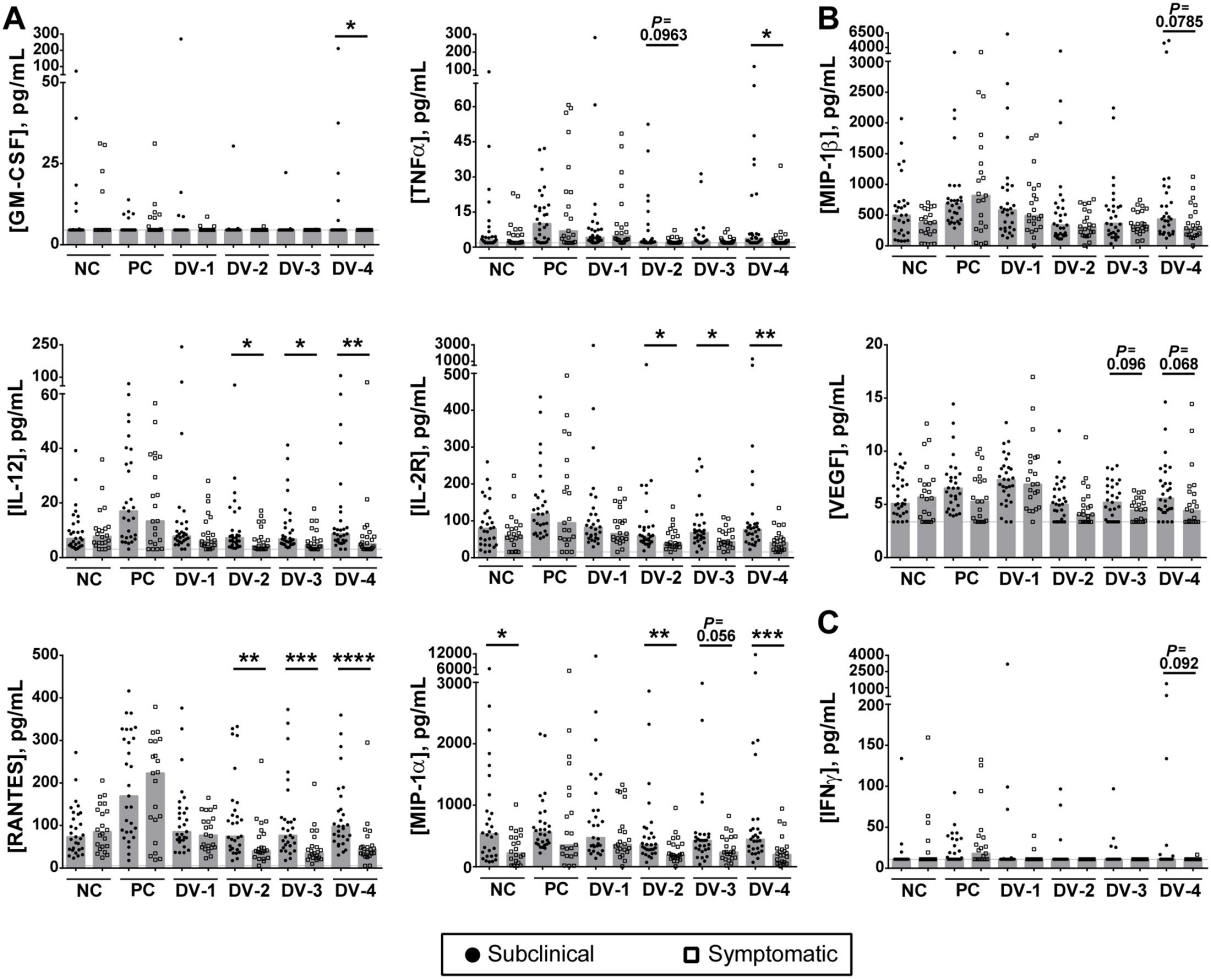
Increased cytokine production in subjects who subsequently had subclinical versus symptomatic infections. (**A**) Levels (pg/mL) of GM-CSF, TNFα, IL-12, IL-2R, MIP-1α, and RANTES were elevated in stimulated PBMC of subjects who subsequently developed subclinical (filled circles), as compared to symptomatic (open squares), DENV infections. (**B**) MIP-1β and VEGF responses also showed slightly elevated production by subclinical cases, although they did not reach statistical significance. (**C**) IFNγ expression was largely found only in subclinical cases. Each symbol represents responses from a single sample following stimulation with uninfected supernatant (unstimulated; negative control, NC), a positive control stimulus (anti-CD3; PC), or live DENV-1 (DV-1), DENV-2 (DV-2), DENV-3 (DV-3), or DENV-4 (DV-4). Median responses are represented by the grey bars. The dotted line indicates the lower level of detection for each analyte. The Wilcoxon rank-sum test was used to compare cytokine production between the subclinical and symptomatic groups (*p<0.05, **p<0.01, ***p<0.001, ****p<0.0001).

Analyzing absolute concentrations of select cytokines, we found that levels of GM-CSF, IL-12, IL-2R, MIP-1α, RANTES, and TNFα were significantly higher in DENV-stimulated culture supernatants from subjects with subclinical versus symptomatic infections (Fig 2). While overall levels were low, GM-CSF and TNFα were significantly elevated after stimulation with DENV-4 by subjects who later experienced subclinical DENV infections. Those subjects also produced more IL-12 and IL-2R than symptomatic subjects, which was significant for DENV-2, DENV-3, and DENV-4 stimulations. RANTES showed a similar pattern, except that subjects with symptomatic infections appeared to down-regulate its expression relative to the negative control. MIP-1α levels appeared naturally higher in subclinical subjects (in the absence of stimulation) and remained so in the presence of DENV-2 and DENV-4 stimulation.

Additional cytokine responses indicated higher levels of DENV-specific expression in subjects with subclinical infection compared to subjects with symptomatic infection, including MIP-1β and VEGF (Fig 2B), although these observations did not reach statistical significance. IFNγ responses were relatively modest in culture supernatants following DENV stimulation; of the responders, however, the majority experienced subclinical infections (Fig 2C).

**Fig 3:**
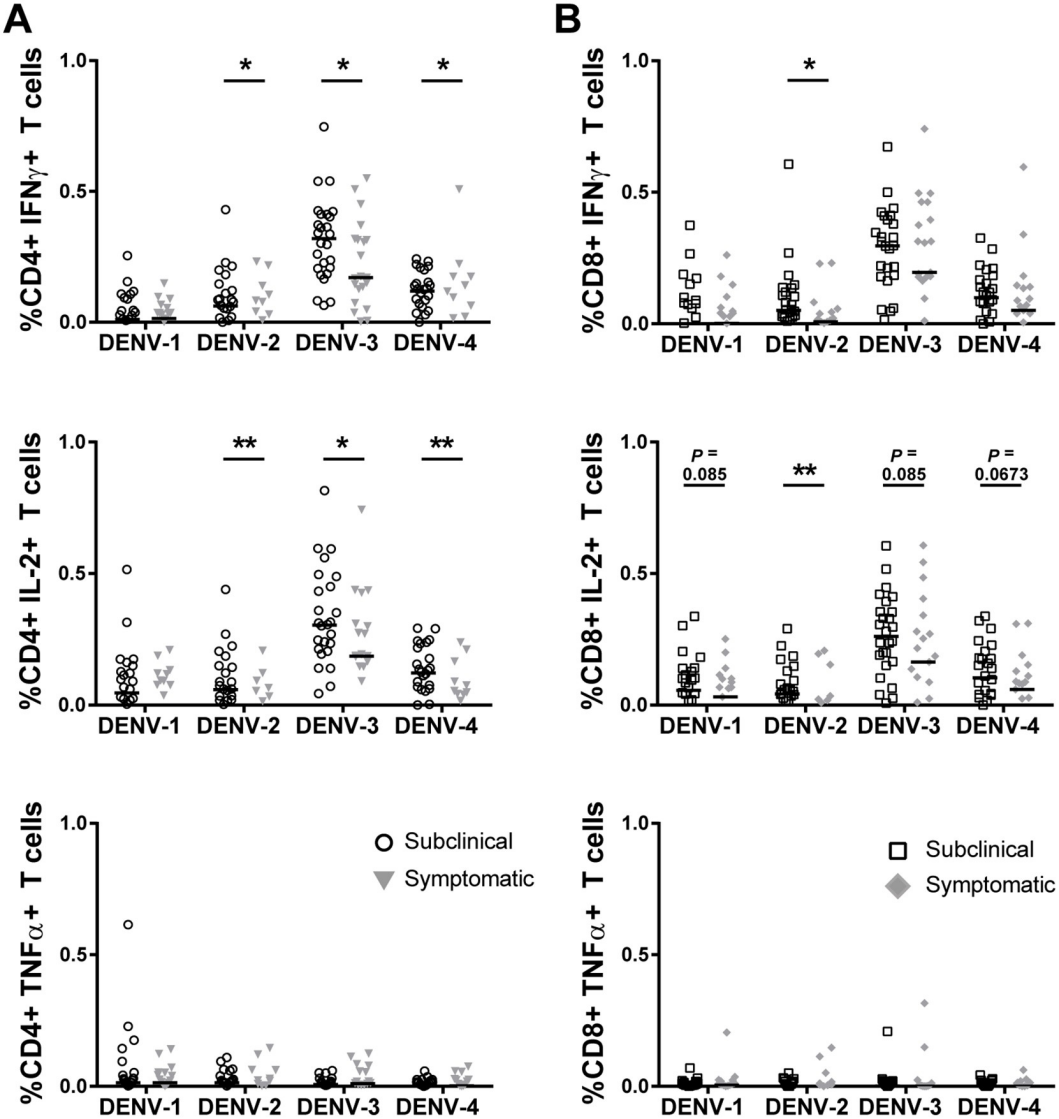
Higher frequencies of DENV-specific T cells in PBMC prior to subclinical versus symptomatic infections. Intracellular IFNγ, TNFα, and IL-2 production by (**A**) CD4+ and (**B**) CD8+ T cells was measured by flow cytometry in response to DENV-1-4 inactivated antigen stimulation of PBMC from Thai schoolchildren. Comparison groups are PBMC from children who later developed subclinical (open symbols) or symptomatic (closed symbols) infections. The Wilcoxon rank-sum test was used to compare cytokine production between the two groups (*p<0.05, **p<0.01). Data are presented relative to the negative control (responses to negative control stimulation were subtracted out).

Previous data from our laboratory indicated significantly higher IFNγ responses in subjects with subclinical infections [9]. As that study used a different assay format, we set up parallel ICS assays using PBMC from the same subjects as used for the cytokine analysis above for comparison. For these experiments, we stimulated PBMC with inactivated antigen overnight and stained for the production of IFNγ, TNFα, and IL-2. Consistent with both the previous study and the trends in cytokine levels noted above, we found higher frequencies of CD4+ and CD8+ T cell producing IFNγ, TNFα, and IL-2 in response to DENV antigen stimulation in the subjects with subclinical infections (Fig 3 and S2 Fig).

### Increased production of IL-6, IL-15, and MCP-1 in subjects who experienced symptomatic DENV infections

**Fig 4:**
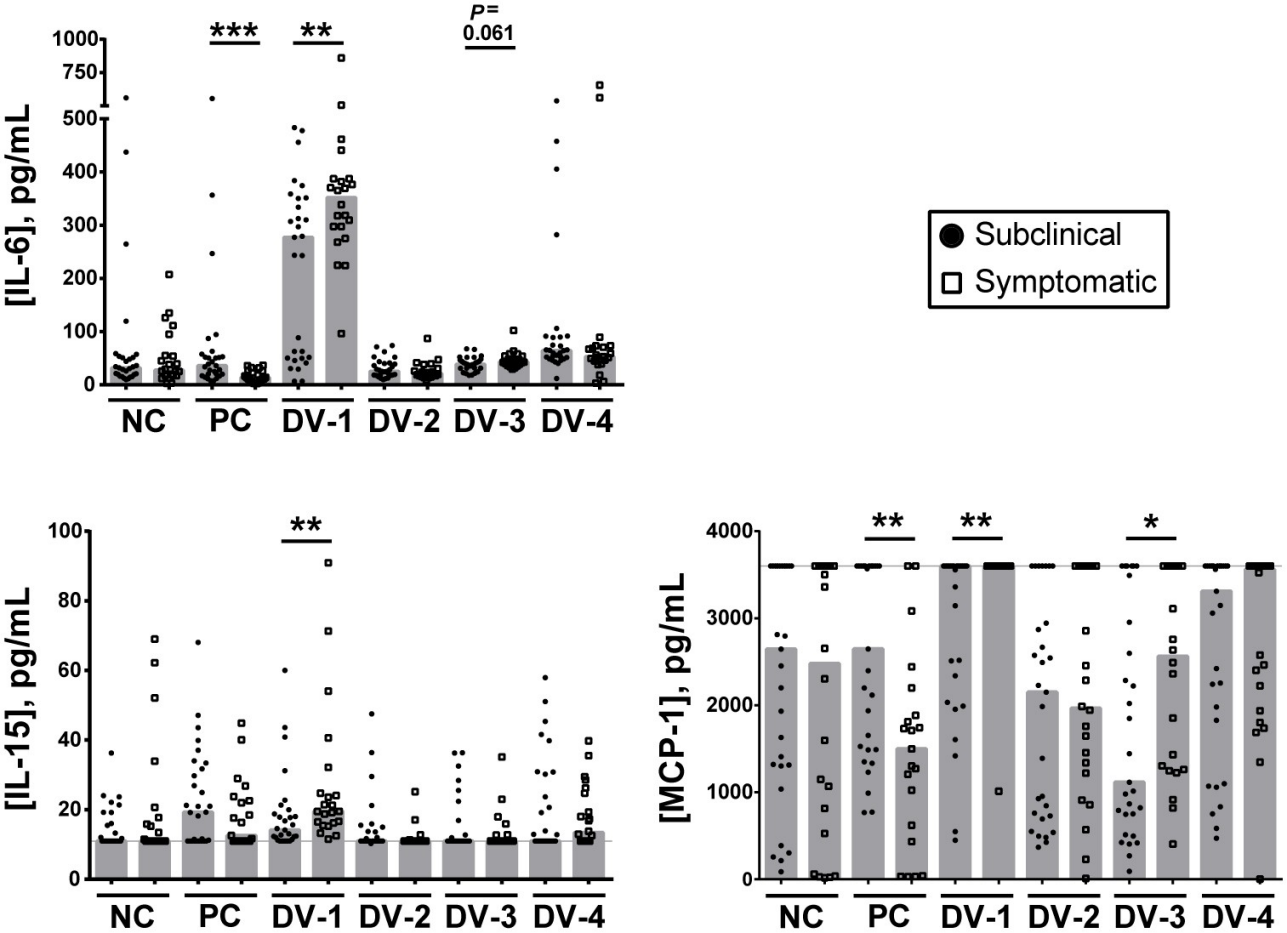
Higher IL-6, IL-15, and MCP-1 production in pre-exposure PBMC from symptomatic, compared to subclinical, cases. IL-6, IL-15, and MCP-1 levels (pg/mL) were higher in culture supernatants of stimulated PBMC from subjects who subsequently developed symptomatic (open squares), as compared to subclinical (filled circles), DENV infections. Each symbol represents responses from a single sample following stimulation with uninfected supernatant (unstimulated; negative control, NC), a positive control stimulus (anti-CD3; PC), or live DENV-1 (DV-1), DENV-2 (DV-2), DENV-3 (DV-3), or DENV-4 (DV-4). Median responses are represented by the grey bars. The dotted line indicates the upper or lower level of detection, as applicable, for each analyte. The Wilcoxon rank-sum test was used to compare cytokine production between the subclinical and symptomatic groups (*p<0.05, **p<0.01).

Levels of IL-6, IL-15, and MCP-1 were higher in response to DENV stimulation in subjects who experienced DF/DHF versus those with subclinical infections (Fig 4). Most striking was IL-6 expression after DENV-1 stimulation. IL-6 production was highly up-regulated in DENV-1 stimulated cultures from all but one symptomatic subject, as compared to 17/29 (58.6%) subjects with subclinical infections (Fig 4). One subject with symptomatic infection and four subjects with subclinical infections who did not produce IL-6 in response to DENV-1 did have a response to DENV-4, and one additional symptomatic subject produced IL-6 in response to both DENV-1 and DENV-4. Neither DENV-2 nor DENV-3 stimulation resulted in substantial up-regulation of IL-6 production, although DENV-3-stimulated wells showed a non-significant trend toward higher levels of IL-6 in subjects with symptomatic infection (p = 0.061).

## Discussion

Through a prospective study in Thailand we identified schoolchildren who experienced subclinical DENV infections, and we compared their pre-existing cytokine profiles to peers who experienced symptomatic infections. Using PBMC samples from approximately 4–7 months prior to DENV exposure, we evaluated the production of 30 different cytokines, chemokines, and growth factors in response to DENV stimulation *in vitro*, modeling interactions that might occur *in vivo* during the subsequent DENV infection. Our analysis revealed that stimulation of PBMC with all four types of DENV induced the production of cytokines, nine of which significantly differed according to the clinical outcome of DENV infection. Six cytokines/chemokines were more highly produced in PBMC of subjects who had subclinical infections, and three cytokines/chemokines were elevated in PBMC of subjects with symptomatic infections. These data identified increased *in vitro* production of IL-12, IL-2R, MIP-1α, RANTES, GM-CSF, and TNFα (possibly along with IFNγ, MIP-1β, and VEGF) as a “protective” immune profile and increased *in vitro* production of IL-6, IL-15, and MCP-1 as a “pathologic” immune profile.

The “protective” profile suggested by our data indicates roles for a robust innate anti-viral response as well as activation of DENV-specific T cells. While we do not know which cell types are responsible for production of these cytokines, the results of the ICS assay indicated that at least some of the TNFα and IFNγ is secreted by T cells. DENV infection of dendritic cells has been demonstrated *in vitro* to induce IL-12 production [13, 14], which presumably contributes to T cell activation. Additionally, the CC chemokines MIP-1α, MIP-1β, and RANTES are known to recruit monocytes/macrophages as well as lymphocytes, supporting a protective role for T cell effectors. These data are in agreement with a study of experimentally DENV-challenged volunteers, which demonstrated higher frequencies of IFNγ-producing T cells in subjects who were protected from disease versus those who were not [15].

Our group previously found increased TNFα production measured by ELISA in response to stimulation with DENV antigen by PBMC collected before infection in children who were hospitalized versus not hospitalized during the subsequent secondary DENV infection [16]. In a later study involving a separate group of subjects, we found higher frequencies of IFNγ- and IL-2–producing T cells measured by ICS staining after overnight stimulation with DENV antigen among schoolchildren who subsequently developed subclinical infection, compared with those who developed symptomatic, secondary DENV infection [9]. In both of the previous studies, we stimulated PBMC *in vitro* with inactivated, DENV-infected Vero cell lysates that was shown to predominantly stimulate CD4+ T cells. In the current study we set up both ICS and 7-day culture assays, the latter using infectious DENV and expanding the number of cytokines/chemokines tested. We found additional chemokines whose production was associated with either subclinical or symptomatic DENV infections. Comparison of data from the two different assays is complicated by the differences in stimulation conditions (infectious virus versus inactivated DENV-infected Vero cell lysate), length of incubation time following stimulation (overnight versus 1 week), and assay method (intracellular cytokine staining versus cytokine analysis of cell culture supernatants); however, compiling the results from this and previous studies suggests anti-viral cytokine production is generally beneficial.

In a human challenge model of influenza, IL-6, IL-15, and MCP-1, among others, were highly upregulated in the first several days post-challenge in subjects who later developed symptoms; while serum samples from subjects with no symptoms post-challenge did not have elevations of these cytokines [17]. Those results are similar to our findings here in the context of natural DENV infection. IL-15 is important for maintaining memory CD8+ T cells and NK cells and is being used in cancer treatments to increase NK cell-mediated anti-tumor activity. In hantavirus infection, however, IL-15-mediated activation of NK cells was shown to circumvent self-tolerance mechanisms leading to targeting of uninfected endothelial cells, demonstrating a potential role in pathogenesis [18]. MCP-1 is involved in recruitment of monocytes/macrophages and lymphocytes and has been implicated in diverting T cell responses from a Th1 toward a Th2 type response [19]. Such a shift in responses would be consistent with the lack of IFNγ production we saw in the current study in symptomatic subjects. Further study of these cytokines and of the role of NK cells in particular in DENV infection is warranted.

The production of IL-6 in PBMC from all symptomatic individuals compared with only a subset of subclinical individuals was a particularly striking finding in this study and suggests its potential role in dengue disease pathogenesis. IL-6 levels have been shown to be elevated in the sera of dengue patients and in several studies were significantly associated with more severe disease [4, 5] IL-6 has various roles in immunity; it has been shown to increase the production of anti-platelet or anti-endothelial cell auto-antibodies and tissue plasminogen activator [20, 21]. *In vitro*, DENV has been shown to induce the upregulation of TLR-2 and TLR-6 which stimulated the production of IL-6 and TNFα in PBMC [22] Recombinant DENV NS1 protein was also shown to stimulate IL-6 production *in vitro* [23]; however, this may not explain completely our finding, as we saw type-specific differences in the induction of IL-6. Identification of the cell subsets responsible for IL-6 production would be an important extension of our study and would further clarify the immunological pathways responsible.

Several limitations of the present study should be noted. We measured cytokine levels at a single time point post-stimulation; assessing protein concentration at earlier and/or later time points to create a kinetic profile of these cytokines might yield additional information. Although we tested a wider array of cytokines compared to our earlier studies, most did not show significant associations with clinical outcome, and many other cytokines were not included in the panel. We also used a single representative virus strain for each DENV type; thus, differences in cytokine production observed between the four types may reflect characteristics of the specific strains used rather than apply generally to that type. Cytokine levels were measured in the cell culture supernatants, and therefore we are not able to identify the cell types responsible for their production. It is possible that some cytokines were secreted in response to initial cytokine secretion by other cells, for example antigen-specific T cells, highlighting the dynamic inter-cellular relationship of cytokine secretion and the resulting complexity underlying interpretation of these data. Additionally, as our study population included very few subjects who were hospitalized, we cannot determine if the “pathologic” immune profile we observed is associated with more severe presentations of dengue. Finally, our cohort study design limited us to PBMC samples from approximately 4–7 months prior to DENV infection. Any changes in immune profiles during that time due to other, related infections or the process of waning immunity are therefore unknown. Future study designs will attempt to close that temporal gap in order to address the influence of an individual’s current immune profile on an incoming infection.

Our results suggest that the type of DENV used for stimulation has a strong influence on cytokine secretion. Although it is difficult to standardize the virus inocula across types, stimulation with DENV-1 appeared to induce a different immune profile compared to stimulation with DENV-2, -3, and -4. In symptomatic individuals, DENV-2, DENV-3, and in some cases DENV-4 appeared to induce down-regulation of chemokine responses while stimulation with DENV-1 increased IL-15, IL-6 and MCP-1 levels significantly. It should be noted that all of the subjects included in the present study experienced DENV infection in 1998 and were in primary school during that year. Thus, the history of prior DENV infections in this study population may be relatively uniform. We do not have data on previously infecting types for these children, and as mentioned above extrapolating such information based on NAb titers is not straightforward. This therefore limits our ability to analyze type-specific results in the context of previous infection history. In other studies using PBMC collected from Thai children with DENV infection we found preferential recognition of the DENV-1 variant of an epitope on the NS3 protein by CD8+ T cells [24], and of DENV-1 virus by B cells [25]. Further studies will therefore be needed to determine if these differences reflect an inherent quality of DENV-1, genetic factors in the Thai population, or the immune history specific to this geographic region and year.

In summary, our analysis of cytokine production by PBMC collected prior to DENV infection in response to DENV stimulation revealed a number of cytokines/chemokines whose production was associated with the clinical outcome of infection. Our findings suggest that profiles of “protective” or “pathologic” cytokines might contribute to an individual having a subsequent subclinical or clinically apparent secondary DENV infection.

## Supporting information

S1 Fig Cytokines at low or undetectable levels in most subjects. PBMC from subjects who went on to experience subclinical (n = 29) or symptomatic (n = 22) DENV infections were stimulated *in vitro* with anti-CD3 antibody (positive control), live DENV-1, live DENV-2, live DENV-3, live DENV-4, or uninfected Vero cell supernatant (negative control). After 6–7 days, culture supernatants were assessed by a multiplexed, bead-based array for quantification of 30 cytokines/chemokines/growth factors. Shown are three- and five-fold changes up (light and dark blue, respectively) or down (grey and pink, respectively), relative to the negative control, of each listed analyte. Each row represents responses from a single individual.
(PDF)

S2 Fig Example of ICS flow plots. The flow cytometry gating strategy to identify cytokine-secreting T cells started with selecting lymphocytes, based on forward and side scatter profiles, followed by singlet cells, dead cell dye exclusion (live cell gate), CD3+ cells, and finally CD4+ or CD8+ T cell subsets. The bottom three rows of plots show CD4+ T cells expressing IFN-gamma, IL-2, or TNF-alpha after no stimulation (negative control) or stimulation with DENV-1 antigen (DENV-1 Ag) or PMA+ionomycin (positive control). Shown are plots from a single representative subject.
(PDF)

S1 Table Additional serological data on the cohort. (PDF)

## References

[1] Endy TP, Anderson KB, Nisalak A, Yoon IK, Green S, Rothman AL, et al Determinants of inapparent and symptomatic dengue infection in a prospective study of primary school children in Kamphaeng Phet, Thailand. PLoS neglected tropical diseases. 2011;5(3):e975 10.1371/journal.pntd.0000975 . 21390158 PMC3046956

[2] Gordon A, Kuan G, Mercado JC, Gresh L, Aviles W, Balmaseda A, et al The Nicaraguan pediatric dengue cohort study: incidence of inapparent and symptomatic dengue virus infections, 2004–2010. PLoS neglected tropical diseases. 2013;7(9):e2462 10.1371/journal.pntd.0002462 . 24086788 PMC3784501

[3] Nimmannitya S, Halstead SB, Cohen SN, Margiotta MR. Dengue and chikungunya virus infection in man in Thailand, 1962–1964. I. Observations on hospitalized patients with hemorrhagic fever. The American journal of tropical medicine and hygiene. 1969;18(6):954–71. . 5355242 10.4269/ajtmh.1969.18.954

[4] Butthep P, Chunhakan S, Yoksan S, Tangnararatchakit K, Chuansumrit A. Alteration of cytokines and chemokines during febrile episodes associated with endothelial cell damage and plasma leakage in dengue hemorrhagic fever. The Pediatric infectious disease journal. 2012;31(12):e232–8. 10.1097/INF.0b013e31826fd456 . 22926216

[5] Guerrero CD, Arrieta AF, Ramirez ND, Rodriguez LS, Vega R, Bosch I, et al High plasma levels of soluble ST2 but not its ligand IL-33 is associated with severe forms of pediatric dengue. Cytokine. 2013;61(3):766–71. 10.1016/j.cyto.2012.12.024 . 23357301

[6] Yong YK, Tan HY, Jen SH, Shankar EM, Natkunam SK, Sathar J, et al Aberrant monocyte responses predict and characterize dengue virus infection in individuals with severe disease. Journal of translational medicine. 2017;15(1):121 10.1186/s12967-017-1226-4 . 28569153 PMC5452397

[7] Endy TP, Chunsuttiwat S, Nisalak A, Libraty DH, Green S, Rothman AL, et al Epidemiology of inapparent and symptomatic acute dengue virus infection: a prospective study of primary school children in Kamphaeng Phet, Thailand. American journal of epidemiology. 2002;156(1):40–51. . 12076887 10.1093/aje/kwf005

[8] Endy TP, Nisalak A, Chunsuttiwat S, Libraty DH, Green S, Rothman AL, et al Spatial and temporal circulation of dengue virus serotypes: a prospective study of primary school children in Kamphaeng Phet, Thailand. American journal of epidemiology. 2002;156(1):52–9. . 12076888 10.1093/aje/kwf006

[9] Hatch S, Endy TP, Thomas S, Mathew A, Potts J, Pazoles P, et al Intracellular cytokine production by dengue virus-specific T cells correlates with subclinical secondary infection. The Journal of infectious diseases. 2011;203(9):1282–91. 10.1093/infdis/jir012 . 21335561 PMC3069729

[10] WHO. Dengue haemorrhagic fever: diagnosis, treament, prevention and control. Geneva: World Health Organization, 1997.

[11] Endy TP, Nisalak A, Chunsuttitwat S, Vaughn DW, Green S, Ennis FA, et al Relationship of preexisting dengue virus (DV) neutralizing antibody levels to viremia and severity of disease in a prospective cohort study of DV infection in Thailand. The Journal of infectious diseases. 2004;189(6):990–1000. 10.1086/382280 . 14999601

[12] Kurane I, Zeng L, Brinton MA, Ennis FA. Definition of an epitope on NS3 recognized by human CD4+ cytotoxic T lymphocyte clones cross-reactive for dengue virus types 2, 3, and 4. Virology. 1998;240(2):169–74. 10.1006/viro.1997.8925 . 9454689

[13] Libraty DH, Pichyangkul S, Ajariyakhajorn C, Endy TP, Ennis FA. Human dendritic cells are activated by dengue virus infection: enhancement by gamma interferon and implications for disease pathogenesis. Journal of virology. 2001;75(8):3501–8. 10.1128/JVI.75.8.3501-3508.2001 . 11264339 PMC114841

[14] Sun P, Celluzzi CM, Marovich M, Subramanian H, Eller M, Widjaja S, et al CD40 ligand enhances dengue viral infection of dendritic cells: a possible mechanism for T cell-mediated immunopathology. J Immunol. 2006;177(9):6497–503. Epub 2006/10/24. . 17056582 10.4049/jimmunol.177.9.6497

[15] Gunther VJ, Putnak R, Eckels KH, Mammen MP, Scherer JM, Lyons A, et al A human challenge model for dengue infection reveals a possible protective role for sustained interferon gamma levels during the acute phase of illness. Vaccine. 2011;29(22):3895–904. Epub 2011/03/30. 10.1016/j.vaccine.2011.03.038 . 21443963

[16] Mangada MM, Endy TP, Nisalak A, Chunsuttiwat S, Vaughn DW, Libraty DH, et al Dengue-specific T cell responses in peripheral blood mononuclear cells obtained prior to secondary dengue virus infections in Thai schoolchildren. The Journal of infectious diseases. 2002;185(12):1697–703. 10.1086/340822 . 12085313

[17] McClain MT, Henao R, Williams J, Nicholson B, Veldman T, Hudson L, et al Differential evolution of peripheral cytokine levels in symptomatic and asymptomatic responses to experimental influenza virus challenge. Clinical and experimental immunology. 2016;183(3):441–51. 10.1111/cei.12736 . 26506932 PMC4750592

[18] Braun M, Bjorkstrom NK, Gupta S, Sundstrom K, Ahlm C, Klingstrom J, et al NK cell activation in human hantavirus infection explained by virus-induced IL-15/IL15Ralpha expression. PLoS pathogens. 2014;10(11):e1004521 10.1371/journal.ppat.1004521 . 25412359 PMC4239055

[19] Deshmane SL, Kremlev S, Amini S, Sawaya BE. Monocyte chemoattractant protein-1 (MCP-1): an overview. Journal of interferon & cytokine research: the official journal of the International Society for Interferon and Cytokine Research. 2009;29(6):313–26. 10.1089/jir.2008.0027 . 19441883 PMC2755091

[20] Huang YH, Lei HY, Liu HS, Lin YS, Chen SH, Liu CC, et al Tissue plasminogen activator induced by dengue virus infection of human endothelial cells. Journal of medical virology. 2003;70(4):610–6. 10.1002/jmv.10438 . 12794725

[21] Rachman A, Rinaldi I. Coagulopathy in dengue infection and the role of interleukin-6. Acta medica Indonesiana. 2006;38(2):105–8. . 16799214

[22] Chen J, Ng MM, Chu JJ. Activation of TLR2 and TLR6 by Dengue NS1 Protein and Its Implications in the Immunopathogenesis of Dengue Virus Infection. PLoS pathogens. 2015;11(7):e1005053 Epub 2015/08/01. 10.1371/journal.ppat.1005053 . 26226614 PMC4520596

[23] Modhiran N, Watterson D, Muller DA, Panetta AK, Sester DP, Liu L, et al Dengue virus NS1 protein activates cells via Toll-like receptor 4 and disrupts endothelial cell monolayer integrity. Sci Transl Med. 2015;7(304):304ra142 Epub 2015/09/12. 10.1126/scitranslmed.aaa3863 . 26355031

[24] Friberg H, Bashyam H, Toyosaki-Maeda T, Potts JA, Greenough T, Kalayanarooj S, et al Cross-reactivity and expansion of dengue-specific T cells during acute primary and secondary infections in humans. Scientific reports. 2011;1:51 10.1038/srep00051 . 22355570 PMC3216538

[25] Woda M, Friberg H, Currier JR, Srikiatkhachorn A, Macareo LR, Green S, et al Dynamics of Dengue Virus (DENV)-Specific B Cells in the Response to DENV Serotype 1 Infections, Using Flow Cytometry With Labeled Virions. The Journal of infectious diseases. 2016;214(7):1001–9. 10.1093/infdis/jiw308 . 27443614 PMC5021233

