# Supplementary figures and images for: Protective versus pathologic pre-exposure cytokine profiles in dengue virus infection
Source: PLoS Negl Trop Dis. 2018 Dec 17;12(12):e0006975. doi: 10.1371/journal.pntd.0006975 (PMC6312351; doi:10.1371/journal.pntd.0006975)

S1 Fig. Cytokines at low or undetectable levels in most subjects.

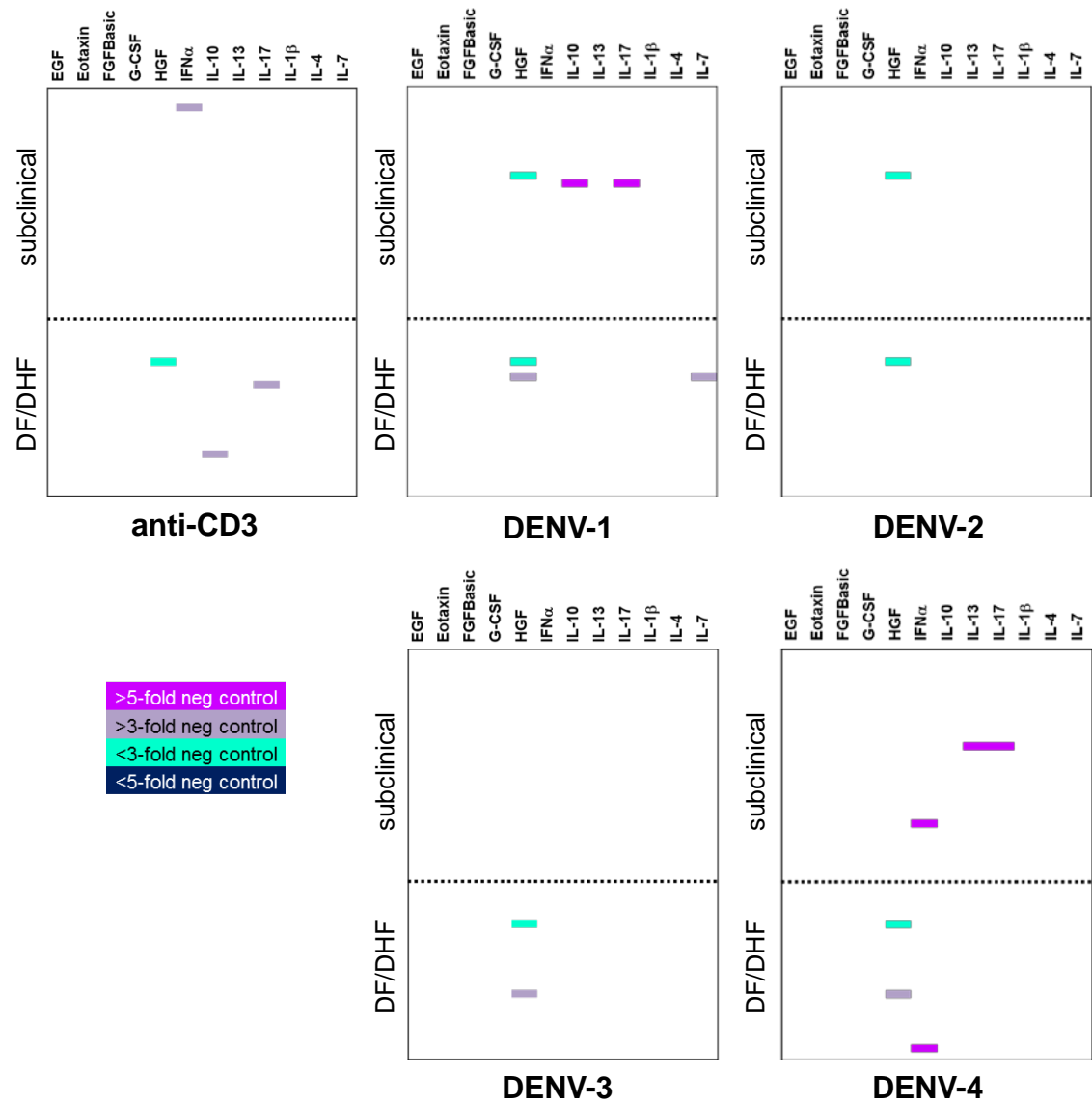

Supplement: S1 Fig — PBMC from subjects who went on to experience subclinical (n = 29) or symptomatic (n = 22) DENV infections were stimulated in vitro with anti-CD3 antibody (positive control), live DENV-1, live DENV-2, live DENV-3, live DENV-4, or uninfected Vero cell supernatant (negative control). After 6–7 days, culture supernatants were assessed by a multiplexed, bead-based array for quantification of 30 cytokines/chemokines/growth factors. Shown are three- and five-fold changes up (light and dark blue, respectively) or down (grey and pink, respectively), relative to the negative control, of each listed analyte. Each row represents responses from a single individual. (PDF) [file pntd.0006975.s001.pdf]

**S2 Fig. Example of ICS flow plots.**

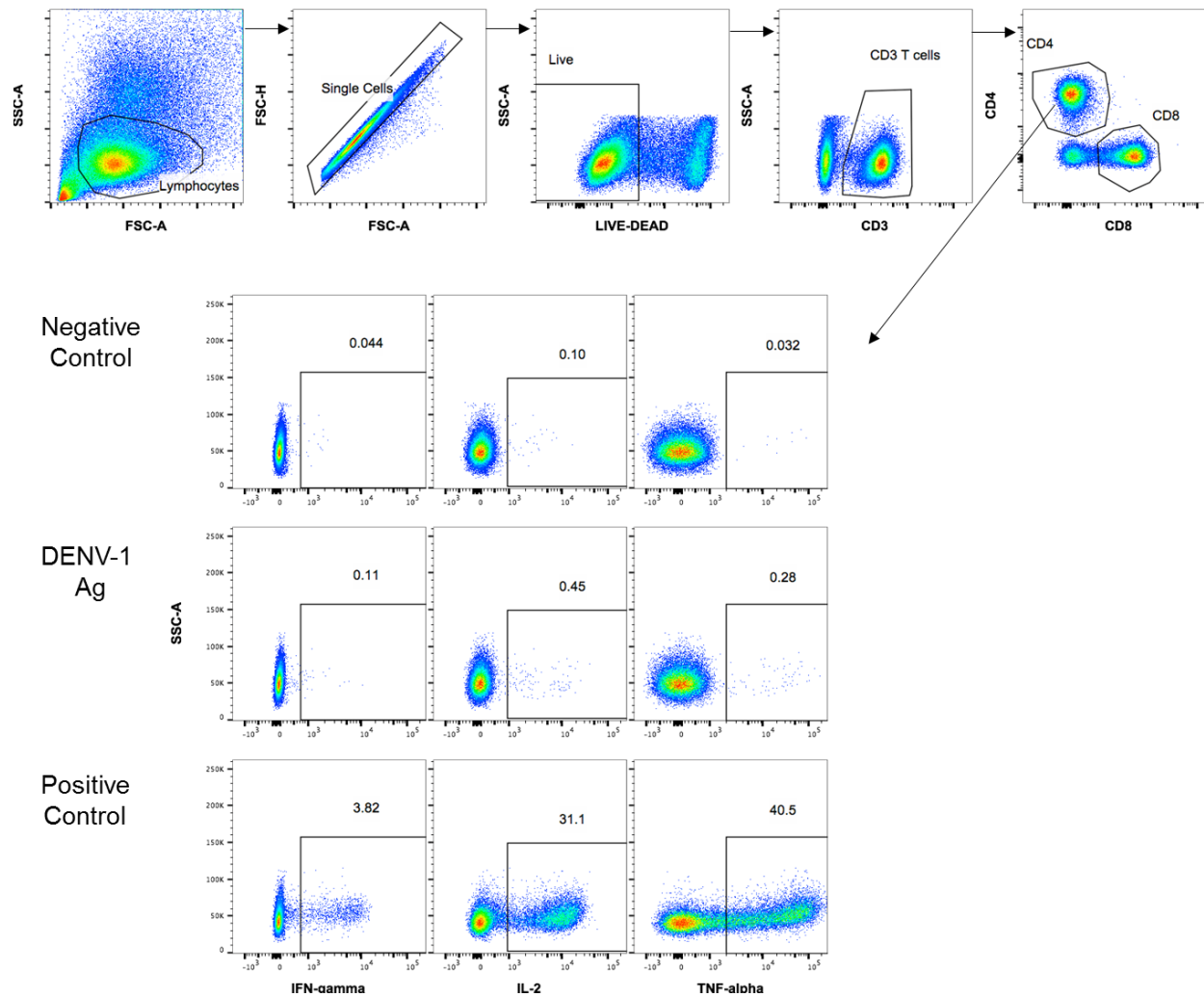

Supplement: S2 Fig — The flow cytometry gating strategy to identify cytokine-secreting T cells started with selecting lymphocytes, based on forward and side scatter profiles, followed by singlet cells, dead cell dye exclusion (live cell gate), CD3+ cells, and finally CD4+ or CD8+ T cell subsets. The bottom three rows of plots show CD4+ T cells expressing IFN-gamma, IL-2, or TNF-alpha after no stimulation (negative control) or stimulation with DENV-1 antigen (DENV-1 Ag) or PMA+ionomycin (positive control). Shown are plots from a single representative subject. (PDF) [file pntd.0006975.s002.pdf]
