# Supplementary material for: Protective versus pathologic pre-exposure cytokine profiles in dengue virus infection
Source: PLoS Negl Trop Dis. 2018 Dec 17;12(12):e0006975. doi: 10.1371/journal.pntd.0006975 (PMC6312351; doi:10.1371/journal.pntd.0006975)
Supplement: S1 Table — (PDF) [file pntd.0006975.s003.pdf]

S1 Table. Additional serological data on the cohort.

Subject No.	Clinical Outcome[a]	PRNT$_{50}$ Titer (pre)[b]				PRNT$_{50}$ Titer (post)[c]				anti-DV EIA Titer (acute)[d]		anti-DV EIA Titer (conv)[e]		Primary or Secondary
		DV-1	DV-2	DV-3	DV-4	DV-1	DV-2	DV-3	DV-4	IgM	IgG	IgM	IgG	
1	Subclinical	<10	161	<10	<10	253	562	181	<10	--	--	--	--	--
2	Subclinical	38	<10	<10	<10	153	404	308	<10	--	--	--	--	--
3	Subclinical	<10	<10	516	<10	>10241	9203	>10241	615	--	--	--	--	--
4	Subclinical	<10	<10	<10	<10	>10241	>10241	>10241	>10241	--	--	--	--	--
5	Subclinical	702	<10	<10	<10	1376	271	228	<10	--	--	--	--	--
6	Subclinical	<10	272	<10	<10	24	386	276	<10	--	--	--	--	--
7	Subclinical	<10	<10	<10	<10	1534	108	2426	<10	--	--	--	--	--
8	Subclinical	<10	<10	<10	<10	254	831	1096	<10	--	--	--	--	--
9	Subclinical	<10	<10	70	<10	401	390	7534	<10	--	--	--	--	--
10	Subclinical	<10	42	<10	<10	166	2245	1811	<10	--	--	--	--	--
11	Subclinical	<10	<10	<10	<10	1941	3751	3751	2651	--	--	--	--	--
12	Subclinical	<10	<10	<10	<10	597	662	239	35	--	--	--	--	--
13	Subclinical	<10	1013	<10	<10	247	3002	4144	<10	--	--	--	--	--
14	Subclinical	<10	171	<10	<10	1903	3751	3437	192	--	--	--	--	--
15	Subclinical	3487	<10	<10	<10	>10241	556	4894	<10	--	--	--	--	--
16	Subclinical	<10	<10	<10	21	293	388	302	59	--	--	--	--	--
17	Subclinical	<10	<10	<10	<10	345	617	2393	<10	--	--	--	--	--
18	Subclinical	<10	<10	<10	<10	131	119	932	<10	--	--	--	--	--
19	Subclinical	<10	261	<10	<10	926	>10241	1641	114	--	--	--	--	--
20	Subclinical	<10	<10	<10	<10	724	5077	3557	28	--	--	--	--	--
21	Subclinical	<10	2449	<10	<10	2107	4005	2699	92	--	--	--	--	--
22	Subclinical	<10	2028	<10	<10	238	1746	1959	<10	--	--	--	--	--
23	Subclinical	<10	<10	<10	<10	259	60	668	<10	--	--	--	--	--
24	Subclinical	<10	<10	<10	30	18	459	545	40	--	--	--	--	--
25	Subclinical	<10	<10	<10	<10	215	508	361	<10	--	--	--	--	--
26	Subclinical	<10	<10	3606	<10	<10	<10	2163	15	--	--	--	--	--
27	Subclinical	167	<10	<10	<10	363	48	485	32	--	--	--	--	--
28	Subclinical	915	<10	<10	<10	1793	<10	95	<10	--	--	--	--	--
29	Subclinical	<10	<10	<10	<10	88	24	199	<10	--	--	--	--	--
30	DF	<10	166	<10	<10	2071	4077	2190	20	0	13	78	178	Secondary
31	DF	<10	152	<10	<10	>10241	7135	3800	57	32	21	53	240	Secondary
32	DF	<10	<10	<10	<10	810	539	780	<10	77	29	92	131	Secondary
33	DF	<10	<10	<10	<10	7756	3477	5492	21	0	9	65	232	Secondary
34	DF	<10	<10	<10	<10	56	32	557	41	4	1	163	118	Secondary
35	DF	415	<10	<10	<10	4593	351	545	<10	53	171	51	133	Secondary
36	DF	2180	<10	<10	<10	5483	605	924	<10	17	225	20	222	Secondary
37	DF	45	<10	<10	<10	535	281	357	<10	41	35	79	242	Secondary
38	DF	<10	<10	<10	<10	1332	402	1428	<10	33	136	21	100	Secondary
39	DF	<10	<10	<10	10	6680	687	4637	380	11	5	89	281	Secondary
40	DF	<10	<10	<10	<10	<10	15	2542	<10	33	15	111	74	Secondary
41	DF	<10	116	<10	<10	1033	1711	2755	<10	12	10	36	185	Secondary
42	DF	<10	<10	<10	<10	810	1085	386	<10	18	27	68	113	Secondary
43	DF	<10	<10	14	<10	2441	431	726	<10	12	4	121	82	Secondary
44	DF	<10	<10	<10	<10	110	432	1006	<10	14	13	64	190	Secondary
45	DF	<10	377	<10	<10	1490	9766	1060	31	1	15	18	155	Secondary
46	DF	<10	<10	<10	<10	>10241	508	7645	<10	9	0	99	154	Secondary
47	DF	<10	161	<10	<10	4139	2370	>10241	128	3	4	13	215	Secondary
48	DF	<10	<10	<10	<10	2671	702	422	<10	183	47	147	62	Primary
49	DHFIII	<10	<10	<10	<10	2017	2336	3005	21	15	10	123	131	Secondary
50	hDF	3999	<10	10	<10	>10241	1555	1495	33	52	6	13	172	Secondary
51	hDF	<10	<10	<10	<10	1597	1589	1798	101	0	5	91	226	Secondary

[a]Subclinical outcomes were defined as seroconversions in the absence of clinically overt illness; symptomatic outcomes were classified according to the 1997 WHO guidelines (refer to Methods)

[b]Prior to the 1998 dengue season (January sample)

[c]After the 1998 dengue season (November sample, except subjects #10, 11, and 14 for whom the June sample was used)

[d]Sample obtained during illness episode

[e]Sample obtained in early convalescence, approximately 2 weeks after acute illness

Abbreviations: DF, dengue fever; DHFIII, dengue hemorrhagic fever grade 3; DV-1-4, dengue virus types 1-4; hDF, hospitalized DF; PRNT, plaque reduction neutralization test
